# A community-based intervention for improving health-seeking behavior among sexual violence survivors: a controlled before and after design study in rural Tanzania

**DOI:** 10.3402/gha.v8.28608

**Published:** 2015-09-25

**Authors:** Muzdalifat Abeid, Projestine Muganyizi, Rose Mpembeni, Elisabeth Darj, Pia Axemo

**Affiliations:** 1 Department of Women's and Children's Health, International Maternal and Child Health (IMCH), Uppsala University, Uppsala, Sweden; 2 Department of Obstetrics/Gynecology, Muhimbili University of Health and Allied Sciences (MUHAS), Dar es Salaam, Tanzania; 3 Department of Epidemiology and Biostatistics, Muhimbili University of Health and Allied Sciences (MUHAS), Dar es Salaam, Tanzania; 4 Department of Public Health and General Practice, Norwegian University of Science and Technology, Trondheim, Norway

**Keywords:** sexual violence, evaluation, community intervention, Tanzania

## Abstract

**Background:**

Despite global recognition that sexual violence is a violation of human rights, evidence still shows it is a pervasive problem across all societies. Promising community intervention studies in the low- and middle-income countries are limited.

**Objective:**

This study assessed the impact of a community-based intervention, focusing on improving the community's knowledge and reducing social acceptability of violence against women norms with the goal to prevent and respond to sexual violence.

**Design:**

The strategies used to create awareness included radio programs, information, education communication materials, and advocacy meetings with local leaders. The intervention took place in Morogoro region in Tanzania. The evaluation used a quasi-experimental design including cross-sectional surveys at baseline (2012) and endline (2014) with men and women aged 18–49 years. Main outcome measures were number of reported rape cases at health facilities and the community's knowledge and attitudes toward sexual violence.

**Results:**

The number of reported rape events increased by more than 50% at health facilities during the intervention. Knowledge on sexual violence increased significantly in both areas over the study period (from 57.3 to 80.6% in the intervention area and from 55.5 to 71.9% in the comparison area; *p*<0.001), and the net effect of the intervention between the two areas was statistically significant (6.9, 95% CI 0.2–13.5, *p*=0.03). There was significant improvement in most of the attitude indicators in the intervention area, but not in the comparison area. However, the intervention had no significant effect on the overall scores of acceptance attitudes in the final assessment when comparing the two areas (−2.4, 95% CI: −8.4 to 3.6, *p*=0.42).

**Conclusions:**

The intervention had an effect on some indicators on knowledge and attitudes toward sexual violence even after a short period of intervention. This finding informs the public health practitioners of the importance of combined strategies in achieving changes.

Despite increasing recognition that sexual violence is a violation of human rights and a major threat to public health, the evidence shows that violence against women is a widespread problem in almost every society (1). Several studies (2, 3) demonstrate how violence against women and girls has a strong potentially deleterious effect on countries’ achievements of at least six of the eight United Nations (UN) Millennium Development Goals (MDGs), and places massive burden on economies of the countries (4). The most recent figures on the extent and nature of violence against women in Tanzania can be found in the demographic health survey of 2010 where at least 20% are reported to have experienced sexual violence in their lifetime (5). According to the World Health Organization, approximately 73 million boys and 150 million girls aged under 18 years worldwide have encountered sexual violence (6).

The world report on violence and health (7) supports the fact that violence is a predictable and preventable health problem. However, there is a need to have effective violence prevention programs that focus on changing individual and community factors (8). In recent years, governments have been increasingly active in implementing policies that may contribute to preventing violence and that strengthen support services for those who experience violence (9). Despite the considerable progress made by African governments on various fronts (e.g. the summit in 2008 facilitated by the Population Council with different sexual and gender-based violence response providers from sub-Saharan African nations), the state of violence in Africa calls for dedicated contribution from various stakeholders, as violence against women and children in Africa continues to be a significant problem throughout the continent (9).

There are a number of community-level interventions focusing on changing the community's attitudes and norms surrounding violence using public information and campaigns. These interventions to address violence against women and girls are ultimately seeking to change deep-seated power relations between men and women. Yet, the central non-negotiable requirement for all interventions across all forms of violence and across all levels of the social ecology has to follow the rule not to harm. Campaigns and media have contributed to a growing awareness of the extent of violence against women (10). The SASA! Study, the first cluster randomized trial in sub-Saharan Africa, evaluates the community-level impact of a gender-focused structural intimate partner violence and HIV prevention intervention. It demonstrates that using community networks, identifying change agents, and applying innovative media with stimulating messages can at least bring intimate partner violence out of the private realm into the public eye (11). Interventions with boys and men demonstrate that addressing unequal gender norms early in life through approaches similar to girls’ life skills programs can influence boys’ and men's perceptions of masculinity and gender norms (12, 13). There is little evidence, however, on what constitutes the most promising services to provide to survivors of intimate partner violence such as sexual violence, although one-stop crisis centers are drawing increasing interest (14).

To date, there has been greater focus on the formal evaluation of interventions using strong research approaches. Still, interventions are few and far between, and most are not evaluated in a manner that can provide promising lessons for replication or scale-up. Key gaps include the lack of evaluated interventions using a strong research design, either experimental or quasi-experimental, with evidence of a significant preventive effect. Rigorous evaluation of any such intervention is limited, particularly in low- and middle-income countries. This study assessed the impact of a community intervention: an awareness creation program, focusing on primary prevention, in order to prevent violence before it occurs; and secondary prevention, the immediate response to violence to improve health-seeking behavior and care among sexual violence survivors.

## Methods

### Study sites

The study was conducted in two neighboring rural districts of southeast Tanzania located approximately 450 km from Dar es Salaam. Kilombero district as the intervention area has 5 divisions, 19 wards, 81 villages, and 365 hamlets, with approximately 416,401 inhabitants (2009 local census). The health system is composed of 1 designated district hospital, 1 private hospital, 5 health centers, and 38 dispensaries. Ulanga district as the comparison area has 7 divisions, 31 wards, 91 villages, and 40 health facilities with 2 hospitals, one of which is a district hospital, with approximately 234,219 inhabitants (2009 local census). These are rural districts separated by Kilombero River. The main economic activities are farming and small-scale fishing. The literacy rate for men is 85 and 73% for women in the whole Morogoro region (5), of which 12% of women and 14% of men had completed some primary level of schooling. All of the health centers and district hospitals in both the districts provide voluntary counseling and treatment for HIV and post-rape care services, though with limited resources.

### Study design and data collection

This was an intervention study which employed a quasi-experimental design (non-equivalent group pretest–posttest). Pre-intervention and post-intervention surveys were conducted in 2012 and 2014, respectively, both in the intervention and comparison areas. The selection of participants and data collection procedures are described in detail elsewhere (15). Through a multi-staged, random sampling technique where only one village in each district was included, a household survey of community members aged 18–49 years using structured questionnaire was conducted in May–June 2012 in the intervention and comparison areas (*n*=1,568) before the intervention started. A household selection form was used to identify eligible members who were then shortlisted, and then using ballot technique one eligible member was selected. The questionnaire included information on the communities’ knowledge and attitudes toward sexual violence, and the socio-demographic factors. A follow-up, cross-sectional survey (*n*=1,551) using the same questionnaire took place in February–March 2014 after 8 months of implementing the intervention. The same households were followed up using the same sampling procedure, although the household member could be different. Ten research assistants (two men and eight women), who were not inhabitants of the study areas, were selected and trained in the use of the questionnaire, the nature of the study, ethical issues related to this study and techniques to conduct such sensitive interviews (16). The local leaders assisted in introducing the research assistants to the household members. In order to ensure the safety and confidentiality of the participants, a maximum of one person per household was selected to complete the survey. At least, three repeat visits were made to households where participants were not available at the time of the first visit. Each interview lasted for 45 min on average, after obtaining verbal informed consent.

### Intervention

An awareness-creation program, aimed to improve the community's knowledge and attitude toward gender-based violence, specifically sexual violence, was carried out. The strategies used to create awareness included radio programs, information, education, and communication (IEC) materials and advocacy meetings with local leaders including religious leaders. The program was implemented only in the intervention area.

### Radio

The radio program was broadcast in the intervention area through the local radio station, Pambazuko FM. This station reaches more than 5 million people who are inhabitants of the Kilombero district and areas of the Ulanga district along its borders, and the Iringa region. The radio sessions covered the following topics: the magnitude of violence against women and children, the laws that convict the perpetrators, the health and social consequences of violence, the importance of seeking care, type of care to expect at health facilities and from police, and the role of the community in supporting sexual violence survivors. A total of four educative sessions were pre-recorded by the first author, MA. Each session was an hour in length and these were broadcast in rotation, once a week for a month, over a period of 8 months. Once every 2 months, there was a live radio program where community members had the opportunity to call in or send SMS and ask questions. MA also facilitated all of the live radio programs.

### IEC and advocacy meetings

Similar topics were also developed into fliers and posters and distributed to the community. A total of 10,000 fliers and posters were produced and disseminated. The majority (7,000 copies) of fliers and posters were disseminated in the surveyed village. The remaining copies were distributed to other health facilities in other divisions. Periodical meetings (every 2 months) were conducted by MA and co-author PM with religious and community leaders to sensitize their unique role in influencing the community's behavior. The meetings were also limited to the village in which household surveys took place. These leaders also received fliers and posters for them to distribute in the community.

This awareness-raising program commenced in May and ended in December 2013 after providing capacity-building training (including medical and psychological treatment of rape survivors, responsibility of health workers, survivor-centered medical history) to health care workers employed at local health facilities. The health facilities were provided with national management guidelines, essential drugs, and other supplies, such as those required for forensic evidence collection. The radio programs ended by urging the community to refer the survivors to the local health facilities for further care and support during and after the end of the intervention.

### Outcome measures

The main outcome measures were the number of reported rape events at the health facilities, and communities’ knowledge and attitude toward sexual violence. A binary outcome variable was created for knowledge and attitude toward sexual violence. The total scores were dichotomized using the two-third rule as described elsewhere (15). Participants who scored ≤66% in either knowledge or attitude were considered as having incorrect knowledge/non-accepting attitudes toward sexual violence, while those who scored 67–100% were considered as having correct knowledge/accepting attitudes toward sexual violence. The program's effect was assessed by comparing the pre-intervention composite score with the post-intervention scores within and between districts.

### Analysis

Data were double-entered using Epidata 3.0 and analyzed using SPSS version 21 and SAS version 9.4 (SAS Institute Inc., Cary, NC, USA). The Chi-square test was used for comparison of demographic characteristics between the intervention and comparison areas, at baseline and endline, except for the variable age where an unpaired *t*-test was used. In the analysis of intervention effect, each question was categorized as correct/incorrect (for knowledge) or acceptance/non-acceptance (for attitude). The aim of the statistical analyses was to estimate the effects of intervention on the percentage of correct knowledge or acceptance attitude answers. The effect of the community intervention was estimated as a net intervention effect (NIE). The NIE was calculated as the difference in intervention area from baseline to endline minus difference in comparison area from baseline to endline with respect to the percentage of correct knowledge or acceptance attitude answers. Since each of the four samples contained a unique set of subjects, the estimated percentages were independent. *P*-values from *z* tests and 95% confidence intervals for the effects were calculated based on a normal distribution assumption. A *p*<0.05 was considered a statistically significant result.

### Ethical clearance

Ethical approval was obtained from Muhimbili University of Health and Allied Sciences (MUHAS) research committee under an umbrella project, ‘Interventions to improve health seeking behavior and care among survivors of Rape and Child Sexual Abuse, Morogoro, Tanzania’. Written permission from each municipal district was obtained. Ethical guidelines for the researching of violence against women approved by WHO/CIOM (17, 18) were followed. Informed verbal consent was taken from each participant before administering the questionnaires, and the participant's name was not disclosed or used for any purpose.

## Results

### Socio-demographic characteristics

At both baseline and final assessment, communities interviewed in the intervention and comparison areas were similar in terms of gender, education status, and radio ownership (Table 1). However, the comparison area had a significantly higher proportion of participants who were single or who had never married in the final assessment than the intervention area (24.9% vs. 19.2%, *p*<0.001).

**Table 1:** Demographic characteristics of communities involved in the intervention and comparison areas, at baseline and endline

Variable	Baseline (2012)			Endline (2014)		
	
	Intervention	Comparison	*p*	Intervention	Comparison	*p*

	*n*=777 (100%)	*n*=791 (100%)		*n*=807 (100%)	*n*=744 (100%)	
Sex						
Male	310 (39.9)	343 (43.4)	0.16	357 (44.2)	323 (43.4)	0.74
Female	467 (60.1)	448 (56.6)		450 (55.8)	421 (56.6)	
Age (in years)						
Mean [SD]	30.7 [7.4]	29.9 [7.8]	0.03	31 [8.6]	29.8 [8.8]	<0.001
Marital status						
Married/cohabiting	708 (91.1)	705 (89.1)	0.19	652 (80.8)	559 (75.1)	<0.001
Single/never married	69 (8.9)	86 (10.9)		155 (19.2)	185 (24.9)	
Education						
No formal education	65 (8.4)	77 (9.7)	0.25	62 (7.7)	59 (7.9)	0.52
Primary	646 (83.1)	662 (83.7)		642 (79.6)	604 (81.2)	
Secondary+above	66 (8.5)	52 (6.6)		103 (12.8)	81 (10.9)	
Occupation						
Peasant/farmer	667 (85.8)	703 (88.9)	0.07	645 (79.9)	633 (85.1)	<0.001
Others	110 (14.2)	88 (11.1)		162 (20.1)	111 (14.9)	
Radio ownership						
Yes	599 (77.1)	612 (77.4)	0.90	624 (77.3)	552 (74.2)	0.15
No	178 (22.9)	179 (22.6)		183 (22.7)	192 (25.8)	

### Participation in the intervention

During the survey in the final assessment, high numbers of community members reported exposure through varied routes: fliers, radio, and meetings with local leaders. Of the 807 individuals interviewed during the final assessment in the intervention area, 51% had received the fliers, 84% heard the radio messages, and 24% heard the messages through the local leaders. However, in the comparison area, of the 744 individuals interviewed, 12% had seen the fliers, 75% heard the radio messages, and 6% heard from the local leaders.

### Reported rape cases

The number of people who had been raped and who attended the health facilities increased from 20 to 55 cases in the intervention area but decreased from 16 to 11 in the comparison area. Of the 55 people who reached the health facilities in the intervention area, all were females and under the age of 18, and only 20 cases arrived on time and received treatment appropriately. The rest arrived after 72 h due to various reasons, such as far distance or lack of money for transport.

### Knowledge on sexual violence

**Fig. 1:**
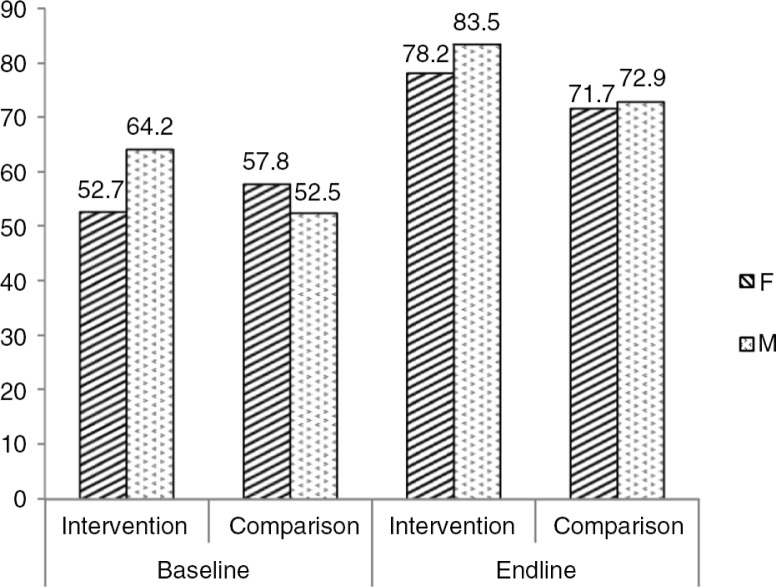
Knowledge on sexual violence by gender of respondents in the intervention and comparison areas, at baseline and endline.

As shown at the bottom of Table 2, the composite scores of knowledge on sexual violence increased significantly in both areas over the study period; from 57.3 to 80.6% in the intervention area and from 55.5 to 71.9% in the comparison area. The intervention had a significant effect in improving awareness of the common perpetrators and services available at the health facilities (*p*<0.001) in the intervention area and the net effect between the intervention and comparison areas was statistically significant (6.9, 95% CI 0.2–13.5, *p*=0.03). When comparing men and women's knowledge, we found a significant difference between men and women at baseline (53% vs. 64%, *p*<0.001) but no significant difference at endline (78% vs. 84%, *p*=0.06) in the intervention area, as shown in Fig. 1.

**Table 2:** Correct knowledge on sexual violence in the intervention and comparison areas at baseline and endline

	Intervention			Comparison					
					
	Pre	Post	Estimate of change	Pre	Post	Estimate of change			
				
	*n*=777	*n*=807	%	*n*=791	*n*=744	%	NIE	95% CI	*p*
Contributing factors of sexual violence									
Effects of alcohol/illicit drugs	585 (75.3%)	662 (82.0%)	6.7	590 (74.6%)	598 (80.4%)	5.8	0.9	−5 to 6.9	0.75
Effects of pornographic films	505 (65.0%)	621 (77.0%)	12	589 (74.5%)	563 (75.7%)	1.2	10.8	4.4 to 17.1	**<0.001**
Changes in our culture	536 (69.0%)	608 (75.3%)	6.4	526 (66.5%)	513 (69.0%)	2.5	3.9	−2.7 to 10.5	0.23
Consequences of sexual violence									
Health and physical effects	661 (85.1%)	739 (91.6%)	6.5	677 (85.6%)	706 (94.9%)	9.3	−2.8	−7.2 to 1.6	0.20
Mental and psychological effects	609 (78.4%)	709 (87.9%)	9.5	634 (80.2%)	652 (87.6%)	7.5	2	−3.3 to 7.3	0.45
Reproductive health effects	634 (81.6%)	727 (90.1%)	8.5	656 (82.9%)	669 (89.9%)	7	1.5	−3.4 to 6.4	0.54
Long-term effect on the victim's development	568 (73.1%)	718 (89.0%)	15.9	635 (80.3%)	646 (86.8%)	6.5	9.3	3.9 to 14.7	**<0.001**
Perpetrators of sexual violence									
Close friends	552 (71.0%)	577 (71.5%)	0.5	615 (77.7%)	476 (64.0%)	−13.8	14.2	7.8 to 20.7	**<0.001**
Close relatives	398 (51.2%)	497 (61.6%)	10.4	463 (58.5%)	399 (53.6%)	−4.9	15.3	8.2 to 22.4	**<0.001**
Sexual Offence Special Provision Act (SOSPA) for Tanzania									
Number of years of imprisonment for perpetrators	684 (88.0%)	722 (89.5%)	1.4	648 (81.9%)	615 (82.7%)	0.8	0.6	−4.3 to 5.7	0.78
Expected services at the health facility									
Contraception	83 (10.7%)	453 (56.1%)	45.5	103 (13.0%)	214 (28.8%)	15.7	29.7	23.9 to 35.5	**<0.001**
HIV/AIDS prophylaxis	205 (26.4%)	526 (65.2%)	38.8	170 (21.5%)	229 (30.8%)	9.3	29.5	23.1 to 35.9	**<0.001**
STI treatment	326 (42.0%)	627 (77.7%)	35.7	255 (32.2%)	459 (61.7%)	29.5	6.3	−0.4 to 13	0.06
Wound care	724 (93.2%)	774 (95.9%)	2.7	724 (91.5%)	681 (91.5%)	0	2.7	−0.9 to 6.4	0.13
Psychotherapy	605 (77.9%)	650 (80.5%)	2.7	572 (72.3%)	573 (77.0%)	4.7	−2	−8 to 4	0.50
Legal verification	689 (88.7%)	752 (93.2%)	4.5	711 (89.9%)	687 (92.3%)	2.5	2.1	−2 to 6.1	0.31
Composite scores									
Correct knowledge	445 (57.27%)	650 (80.55%)	23.3	439 (55.5%)	535 (71.91%)	16.4	6.9	0.2 to 13.5	**0.03**

CI, confidence interval; NIE, net intervention effect (difference in intervention area from baseline to endline minus difference in comparison area from baseline to endline). Significant values are provide in bold.

### Attitudes toward sexual violence

**Fig. 2:**
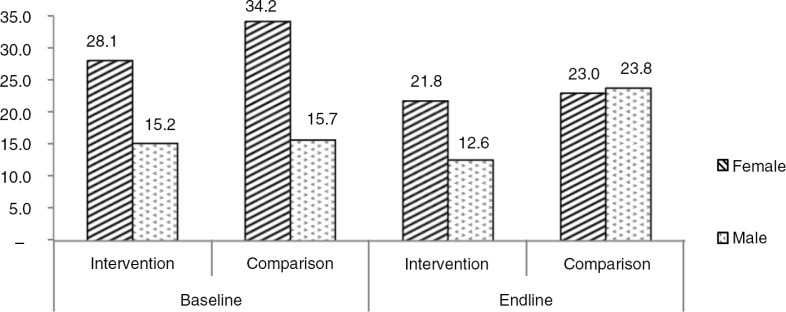
Acceptance attitudes toward violence against women norms by gender of respondents in the intervention and comparison areas at baseline and endline.

There was a significant reduction in most of the attitude indicators favoring male dominance, justification of men beating their partners, as well as rape myths, in the intervention area, but not in the comparison area (Table 3). However, the intervention had no effect in the final assessment when comparing the two areas (−2.4, 95% CI: −8.4–3.6, *p*=0.42). There was a reduction in acceptance attitudes toward violence against women norms, with a significant difference between men and women at baseline (*p*<0.001) as well as at final assessment (*p*<0.001) in the intervention area, as shown in Fig. 2.

**Table 3:** Acceptance of violence against women between intervention and comparison areas at baseline and endline

	Intervention			Comparison					
					
	Pre	Post	Estimate of change	Pre	Post	Estimate of change			
				
	*n*=777	*n*=807	%	*n*=791	*n*=744	%	NIE	95% CI	*p*
Attitudes favoring male dominance									
A man should show he is head of household	753 (96.9%)	713 (88.4%)	−8.6	741 (93.7%)	694 (93.3%)	−0.4	−8.2	−11.8 to 4.6	**<0.001**
A decent wife obeys her husband	534 (68.7%)	486 (60.2%)	−8.5	622 (78.6%)	565 (75.9%)	−2.7	−5.8	−12.2 to 0.6	0.07
A wife is obliged to have sex with her husband	451 (58.0%)	349 (43.2%)	−14.8	561 (70.9%)	395 (53.1%)	−17.8	3	−3.9 to 10	0.38
Marital disputes should not be exposed outside	701 (90.2%)	718 (89.0%)	−1.2	711 (89.9%)	671 (90.2%)	0.3	−1.5	−5.9 to 2.8	0.47
Husband disciplines the wife by beating her	580 (74.6%)	696 (86.2%)	11.6	579 (73.2%)	629 (84.5%)	11.3	0.3	−5.5 to 6	0.93
Opinions on justifying men beating his wife/partner									
Reason to hit: wife/partner does not fulfill household duties	298 (38.4%)	229 (28.4%)	−10	302 (38.2%)	292 (39.3%)	1.1	−11	−17.9 to −4.2	**<0.001**
Reason to hit: wife/partner refuses sex	273 (35.1%)	195 (24.2%)	−11	206 (38.1%)	180 (33.5%)	−4.6	−6.4	−13.1 to 0.3	0.06
Reason to hit: wife/partner opposes his views/opinions	223 (28.7%)	158 (19.6%)	−9.1	259 (32.7%)	203 (27.3%)	−5.4	−3.7	−10 to 2.7	0.23
Reason to hit: wife/partner is unfaithful	345 (44.4%)	232 (28.7%)	−15.7	368 (46.5%)	377 (50.7%)	4.2	−19.9	−26.8 to −12.8	**<0.001**
Reason to hit: wife/partner is alcohol/drug abuse	157 (20.2%)	142 (17.6%)	−2.6	301 (26.0%)	249 (24.2%)	−1.8	−0.8	−6.7 to 5.2	0.80
Reason to hit: wife/partner insults/disrespects	327 (42.1%)	224 (27.8%)	−14.3	327 (41.3%)	362 (48.7%)	7.4	−21.7	−28.6 to −14.7	**<0.001**
Rape myths − reason women and girls are raped is because of									
the way they dress or act	110 (14.2%)	314 (38.9%)	24.8	199 (25.2%)	466 (62.6%)	37.4	−12.7	−19.1 to 6.4	**<0.001**
the place they work (bar, clubs, prostitute)	509 (65.5%)	229 (28.4%)	−37.1	536 (67.8%)	311 (41.8%)	−26	−11.2	−17.9 to 4	**<0.001**
they walk alone at night	343 (44.1%)	266 (33.0%)	−11.2	394 (49.8%)	312 (41.9%)	−7.9	−3.3	−10.3 to 3.7	0.35
Composite scores									
Accepting attitude	178 (22.9%)	143 (17.2%)	−5.2	207 (26.2%)	174 (23.4%)	−2.8	−2.4	− 8.4 to 3.6	0.42

CI, confidence interval; NIE, net intervention effect (difference in intervention area from baseline to endline minus difference in comparison area from baseline to endline). Significant values are provide in bold.

## Discussion

In this controlled study, we attempted to evaluate a community-based intervention to improve health-seeking behavior. The number of rape survivors who reported to the health facilities increased by more than 50%. The intervention had an effect not only on awareness of the health consequences of sexual violence and services available in the health facilities, but also some indicators of social acceptability of violence against women norms.

Significant positive effects of the intervention were noted on the knowledge of health consequences and the treatments available at the health facilities, with no differential effect on gender. However, there were also similar improvements in the comparison area during this time period. These improvements may be due to other exogenous efforts. Many non-governmental organizations (NGO) in Tanzania have launched mass media and legal literacy campaigns to raise awareness of GBV and to encourage men to be role models in confronting violence (13). One example is the Fataki Campaign, which aired in Tanzania *via* radio from 2008 to 2011, aimed to incite social disapproval of men who engage in cross-generational sex, and to encourage community interventions in these relationships through social learning (19). It was also reported that 75% of participants in the comparison group were also exposed to radio messaging. This is a high proportion and could have had a dilution effect on differences between groups. Nonetheless, in the current intervention evaluated in this study, increased knowledge in the community related to sexual violence was not sufficient enough to increase the reporting of rape events to the health facilities in the comparison area. On the other hand, in the intervention area, where the health care workers received training and were provided with material resources and guidelines, thus assuring adequate medical services for survivors, there was an increase in the number of rape survivors who reported and sought care at the health facilities. However, the fact that all reported rape cases were under 18 years of age, confirms what was previously found that for an adult survivor besides significant financial, structural, and infrastructural barriers, social barriers play a major role in care-seeking (20). Therefore, in addition to comprehensive health services and care, it is important for any health development program that wishes to successfully respond to sexual and gender-based violence to also incorporate approaches to tackle social barriers that hinder care-seeking and justice for survivors.

The intervention also focused on violence prevention through social norm change at the community level. There was a significant reduction in some indicators of violence acceptance, such as attitudes favoring male dominance, justification of men beating their partners, and rape myths. Similar findings have been revealed in the evaluation of the ‘Stepping Stone’ intervention study at different sites (12). Specifically, the ‘Stepping Stone’, a community-based participatory training program, reduced the social acceptability of wife-beating at the community level. Although the overall net effect was not significant in the current study, it has been noted that increased sensitization to the issue may have led to decreased reporting of negative behaviors and over-reporting of favorable attitudes in the intervention and comparison areas (17). Perhaps, social desirability bias may have influenced our results, but at least this indicates a positive shift in perceived social norms, taking into account the short period of the intervention. Moreover, the intervention used gender neutral approach, but we demonstrated differential effects of intervention on gender. There was reduction but with significant difference between men and women violence acceptance attitudes, unlike in previous studies which did not show any differential effect on gender using the same approach (21). As observed in the SASA! Study, change at the community level is possible by engaging with both men and women at all levels, and by explicitly focusing on power rather than gender (11), but this requires sustained effort and a commitment to a human rights perspective.

Furthermore, findings from this study do suggest that gender norms still exists as communities support a man to be the head of the household, and that he could discipline his partner by beating her. They also continue to embrace the rape myth; that is, rape occurs because of the way girls or women dress. Violence against women and girls is highly gendered, and Connell (22) describes gender as relational social practices within our daily life, and places at the top hegemonic masculinity, that is, practices of masculinities that are more socially idealized and associated with social powers than others. Hegemonic masculinities are maintained by cultural consent, and the subordinate status of women helps sustain it. Most men do benefit through economic power, authority, and access to institutionalized power. This means that many men have both physical and financial advantage over their female partners, leaving women more vulnerable to violence. On a societal level, power imbalances with gender norms supporting male dominance in families facilitates violence against women (23). It is obvious that gender permeates all levels of the ecological model; therefore, it is important to take into consideration gender aspect when considering violence and the impact violence has on those not directly exposed.

An important strength of this study is the use of a quasi-experimental study design that included a comparison area and two time-period assessments (pre- and post-intervention) to recognize the temporal trends. Significant change for some knowledge and attitude indicators was apparent in a relatively short period, and if the intervention is sustainable, it is likely that the effects will be even greater. The measures of effect and confidence intervals for all variables (Table 2 and 3) are presented to allow the readers to judge the strength of the evidence for themselves. Although many of these results were significant at individual variable level but not net effect, in addition to significance tests, it is emphasized that when evaluating complex interventions with multiple outcomes, it is important to take into account the directionality, consistency and congruency of observed outcomes (24). However, the study design, with a comparison between two cross-sectional studies, is not as strong as a randomized trial. The intervention and comparison areas were similar despite the huge geographical buffer (approximately 200 km). The communities matched, especially in their education level and radio ownership, which would have implications on knowledge. Although the comparison area was not exposed directly to the intervention, a possible contamination could have occurred due to greater mobility of people across the region. Due to ethical reasons, those aged less than 18 years were not sampled but were the only ones reporting rape to health facilities. It is not known whether they were exposed to the intervention. Children less than 18 years are still under the care of their parents. So if parents’ knowledge increased as a result of the intervention, it is likely that they will report the rape cases for their children. The study design was unable to determine the route that increased rape reporting: knowledge increased in both groups, the intervention did not significantly change attitude. Is the increased rape reporting due to increased knowledge or changed attitudes in health care providers? Or to other contextual factors influencing the behavior of the intervention group not related to the intervention? It is therefore difficult to attribute the increase in rape reports solely to the intervention.

## Conclusions

This is the first evaluation study linked to survivors of sexual violence and their health-seeking patterns to be completed in Tanzania. The study showed that the awareness-creation program used to improve knowledge and attitudes toward sexual violence has an effect on some indicators even after a short intervention (8 months). This finding informs the public health practitioners of the importance of implementing combined strategies in achieving change. Among the lessons learned in the implementation of this program were that a central challenge in sexual violence response is assuring adequate medical services for survivors, and financial support to access justice. Coordinating multiple sectors, from medical, legal, and psychological services, was difficult. It is important for decision-makers to identify ways to have programs disseminated and adopted in different settings which, in this study, has proved to be a great challenge. Further research is needed to understand the promising results of this intervention before the program can be replicated.
